# Ultra-high performance liquid chromatography-size exclusion chromatography (UPLC-SEC) as an efficient tool for the rapid and highly informative characterisation of biopolymers

**DOI:** 10.1016/j.carbpol.2018.05.049

**Published:** 2018-09-15

**Authors:** Natalia Perez-Moral, Jean-Michel Plankeele, Claire Domoney, Frederick J. Warren

**Affiliations:** a Food and Health Programme, Quadram Institute Biosciences, Norwich, NR4 7UA, UK; b Waters S.A.S., BP 608, 78056 Saint-Quentin, En Yvelines Cedex, France; c Department of Metabolic Biology, John Innes Centre, Norwich Research Park, Colney, Norwich, NR4 7UH, UK

**Keywords:** Starch, UPLC, Size exclusion chromatography, Molecular structure, Chain length distribution

## Abstract

Starch has a complex molecular structure, with properties dependent on the relative chain lengths and branching structure of its constituent molecules, which varies due to polymorphisms in starch biosynthetic genes, as well as environmental factors. Here we present the application of ultra-high performance size exclusion chromatography to the separation of starch chains from plant seeds. Several methods, have been used to analyse chain length distributions in starch, all with limitations in terms of analysis time, sample preparation and molecular weight range. Here we demonstrate that chain length distributions can be obtained with dramatically reduced analysis time using ultra-high performance size exclusion chromatography. The method may also show improvements in resolution of some fine structural features. Understanding links between starch fine structure and biosynthetic genes will allow bioengineering of starches with tailored properties. This technique may have application to the size separation and resolution of a range of biopolymers of value to the food, drink and pharmaceutical industries.

## Highlights

• UPLC-SEC used as a novel tool for biopolymer characterisation.
• Faster analysis time and higher resolution than existing techniques.
• Potential for high throughput screening of starch fine structure.

## Graphical abstract

**Figure:**
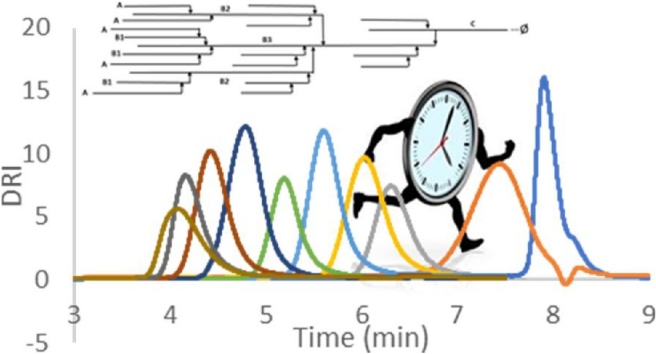

## 1 Introduction

Starch is one of the most important biopolymers on earth, forming the main source of energy in the human diet, as well as being an important feedstock for a range of industrial processes, from the food industry to biofuels and paper manufacture. Starch comprises two distinct biopolymers, amylose and amylopectin. Amylose is an essentially linear molecule formed from α–1 → 4- linked anhydroglucose residues with a molecular weight of 10^5^–10^6^ Da. Amylopectin is a far more complex molecule formed from short chains of α–1 → 4- linked anhydroglucose residues interspersed with α–1 → 6 branch points. These two polymers make up the complex, hierarchical structure of the starch granule. The chain lengths, degree of branching and position of the branches within the molecule are all dictated by a complex system of biosynthetic enzymes including elongating starch synthases, starch branching enzymes, debranching enzymes and amylolytic enzymes. Plants possess several isoforms of each of these biosynthetic and degradative enzymes, which can play subtly different roles and act in concert to generate the final complex structure of the starch molecule.

**Fig. 1:**
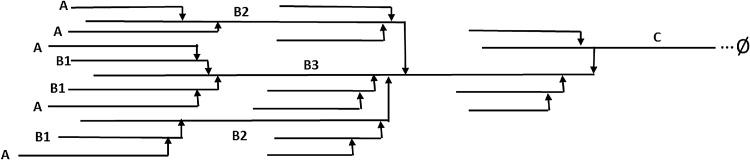
A schematic of the cluster model of amylopectin structure. Adapted from (Hizukuri, 1986).

The most widely measured structural feature of starch polymers is the chain length distribution (CLD), which is the fundamental structural level of starch which determines a range of functionalities such as crystallinity, texture properties and digestion (Witt, Doutch, Gilbert, & Gilbert, 2012). To obtain this distribution, the starch is solubilised and treated with an enzyme (isoamylase or pullulanase) that cleaves the α–1 → 6 branch points, leaving linear chains of varying lengths. These chains can then be separated and quantified. This yields a characteristic distribution, which relates to the structure of the amylopectin and amylose molecules. Within the classic cluster model of amylopectin, the chains of the molecule can be classified by their branching and lengths. Each amylopectin molecule contains only a single C chain, which holds the sole reducing end of the molecule. B chains branch off another chain, and themselves have chains branching from them. B3 chains are the longest, and span multiple clusters, with a chain length >50 glucose residues. B2 chains span more than one cluster and are of intermediate length, 25–50 residues. B1 chains are the shortest, with a length of <25, similar in length to A chains which are the shortest, unbranched chains (Hizukuri, 1986). A schematic of this model of starch structure is shown in Fig. 1. Amylose chains are much longer (100–10,000 glucose residues in length), and their fine structure has received less attention, primarily due to the inherent technical difficulties in characterising such large molecules. The relative lengths of the amylopectin chains have important implications for the structure of the native starch granule with longer chains in the amylopectin clusters more able to form double helices, resulting in higher granule crystallinity and wider crystalline lamellae regions (starch granule structure being composed of alternating crystalline and amorphous layers, termed lamellae) (O'Sullivan & Perez, 1999; Pfannemüller, 1987; Witt et al., 2012). CLDs of both amylopectin and amylose molecules also have important implications for end use functionality and starch properties, determining a wide range of different parameters including gelatinisation properties (Witt et al., 2012), brewing quality (Gous, Warren, Mo, Gilbert, & Fox, 2015), starch digestibility (Syahariza, Sar, Hasjim, Tizzotti, & Gilbert, 2013) and food sensory perception (Li, Prakash, Nicholson, Fitzgerald, & Gilbert, 2016). Analysis of CLDs has also become an important tool to understand the links between starch biosynthetic enzymes and starch structure in order to bioengineer starches with tailored properties, and to better understand starch biosynthesis (Carpenter et al., 2017; Cuevas, Daygon, Morell, Gilbert, & Fitzgerald, 2010; Wu, Morell, & Gilbert, 2013).

Several different techniques have been used in attempts to characterise the CLD of the starch granule, which poses particular challenges in polymer characterisation due to the broad molecular weight distributions involved, and the fine structural variations between different CLD’s. The major separation techniques used include Fluorophore Assisted Capillary Electrophoresis (FACE) (Morell, Samuel, & O'Shea, 1998), HPLC-based Size Exclusion Chromatography (HLPC-SEC) (Ciric, Woortman, & Loos, 2014; Vilaplana, Hasjim, & Gilbert, 2012) and asymmetric field flow fractionation (AF^4^) (Chiaramonte, Rhazi, Aussenac, & White, 2012). HPLC-SEC is the most widely used technique, but it has several technical problems, in particular the extended run times required to separate the very broad range of molecular weights in the starch CLD, which leads to lengthy sample analysis times. HPLC-SEC also suffers from band broadening as a result of low plate counts which result in low efficiency and therefore inherently poor resolution. This loss of resolution is solved in FACE, which does not suffer from band broadening. However, FACE is limited to, at best, a degree of polymerisation (D.P.) of approximately 140, meaning it cannot be used to analyse amylose chain distribution. (Wu et al., 2013) In theory, these limitations are overcome by the use of AF^4^, but in practice it has been found difficult to achieve the required resolution to identify fine structural features using AF^4^ (Chiaramonte et al., 2012; Gidley et al., 2010).

Over the last two decades, the field of liquid chromatography has been revolutionised by the introduction of Ultra-high Perfomance Liquid Chromatography (UPLC), which utilises very high pressures of 40 MPa and above, and column packing materials with a particle size of sub–2 μm (MacNair, Lewis, & Jorgenson, 1997). The use of sub–2 μm columns provides greater separation efficiency, and permits higher linear velocities which reduces analysis times (Swartz, 2005). However, the use of UPLC technology with SEC has been limited, as SEC columns require large, defined pore diameters, which are a challenge to obtain with packing materials that will be stable under UPLC conditions. Only recently have sub–2 μm packing materials been developed for SEC that have the requisite solvent stability and mechanical strength (Uliyanchenko, Schoenmakers, & Van der Wal, 2011). These systems have been shown to provide significant improvements in separation efficiency and resolution of synthetic polymers (Bouvier & Koza, 2014; Janco, Alexander, Bouvier, & Morrison, 2013). In this paper, we describe the application of a UPLC-SEC approach to the separation of starch CLDs. It is anticipated that, by demonstrating the applicability of UPLC-SEC to the more rapid chain length separation of starches, this methodology will find wider use in biopolymer separation and characterisation, as well as becoming an important tool for starch structural analysis. We demonstrate that starch CLDs can be obtained, covering the size distribution range from 6 to 10,000 D.P., identifying features in the amylopectin CLD which are normally only possibly observed in a FACE distribution, and revealing structural heterogeneity in the amylopectin region.

## 2 Materials and methods

Three different starches were used in this study. Barley (*Hordeum vulgare*) (cv. Tipple) was provided as a gift from Phil Howell (NIAB, UK). The grain sample was ground to a flour in an IKA MF 10 mill to pass a 150 μm mesh and starch was extracted from the flour following the method of Andersson, Andersson, and Åman (2001). Pea (*Pisum sativum*) plants were grown at the John Innes Centre (UK), using two smooth-seeded pea cultivars, BC1/19RR (derived from introgression of the wild-type *SbeI* (*starch-branching enzyme I*) allele into the wrinkled-seeded genotype, JI 430) and cv. Cameor. Pea seeds were manually dehulled and then ground in a coffee grinder to pass a 150 μm mesh. From all three samples the starch was then solubilised and debranched as described in Wu, Li, and Gilbert (2014). Pullulan standards with peak molecular weights ranging from 342 to 708,000 Da (PSS-pulkit) were obtained from Kromatek (UK). All samples and standards were dissolved in DMSO containing 0.5% (w/v) LiBr prior to analysis at a concentration of 1 mg/mL. All samples and standards were analysed using a Waters ACQUITY Advanced Polymer Chromatography system fitted with 3 columns in series; XT-450 Å, XT-125 Å and XT-45 Å and a differential refractive index (DRI) detector. The flow rate was set to 0.3 mL/min and the column temperature was 90 °C.

Pullulan standards were used for calibration, using the method described by Cave, Seabrook, Gidley, and Gilbert (2009) to obtain a relationship between elution volume and hydrodynamic radius (V_h_) for the linear glucans, based on Mark-Houwink parameters of K = 0.0002427 dL g^−1^ and α = 0.6804 for pullulan in DMSO at 90 °C. Using this calibration, DRI elution profiles of the debranched starch samples were converted to SEC weight distributions expressed as w(logV_h_) using the relationship:

$$w\left(logV_{h}\right)=S_{DRI}\left(V_{el}\right)\frac{dV_{el}}{dlogV_{h}}$$

where V_el_(V_h_) is obtained from the calibration curve of the pullulan standards (Cave et al., 2009). CLDs were normalised using standard normal variate normalisation. For linear polymers, such as debranched starch, a number distribution (*N_de_(X)*) can be obtained from the corresponding weight distribution obtained from the DRI signal through the relationship:

$$w\left(logX\right)=X^{2}N_{de}\left(X\right)$$

## 3 Results and discussion

**Fig. 2:**
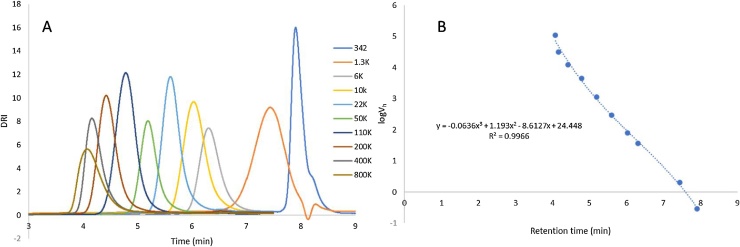
Example elution profiles for pullulan standards (A) and example standard curve (B) derived from the retention times of the pullulan standards.

DRI responses for each of the standards and the associated standard curve are shown in Fig. 2. Separation of the standards was achieved in under 8 min, with good resolution of each standard. A direct comparison of the same standards using HPLC-SEC is shown in Fig. S1, demonstrating that the same separation requires 45 min analysis time. From these standards, a standard curve was constructed, giving an upper limit of calibration of radius of hydration (Rh) ≤ 30 nm. This covers the amylopectin region of the size distribution, and a proportion of the shorter amylose chains. It should be noted that beyond this calibration, an extrapolation of the standard curve is required, and the method should only be considered semi-quantitative for longer amylose chains.

**Fig. 3:**
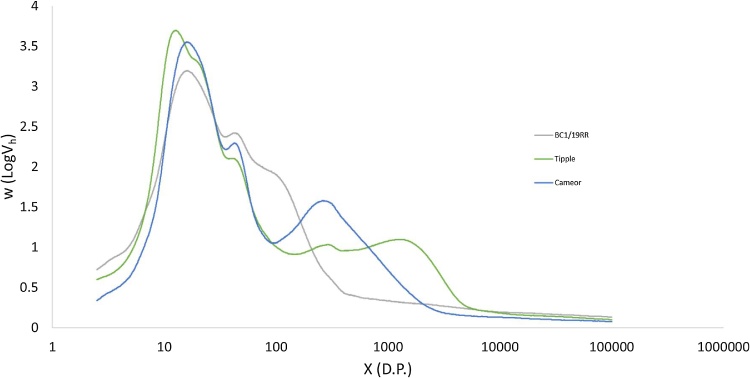
Chain Length Distributions for three different starches expressed as weight distributions as a function of degree of polymerization.

**Fig. 4:**
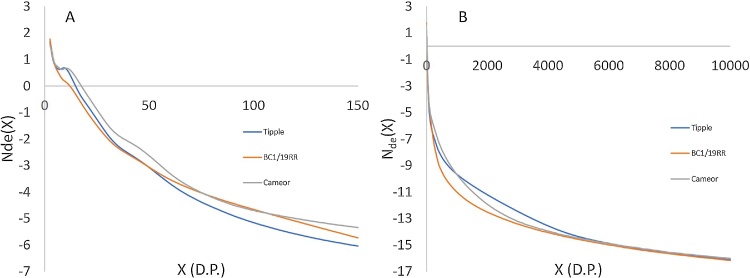
Number distributions derived from DRI traces for three starches. Focusing on the amylopectin region up to D.P. 150 (A) and covering the whole size distribution (B).

Fig. 3 shows weight distribution CLDs for three different types of starch, two different pea genotypes and a common commercial barley variety. Each distribution is eluted in under 8 min, with a total analysis time per sample of ≤15 min. The resolution of structural features within the CLD is equal to, or greater than, that achieved with conventional HPLC-SEC. Within the amylopectin region, both pea starches show two distinct peaks, with the global maximum of the distribution at D.P. 16 and a second amylopectin peak appearing at D.P. 44. The peak at D.P. 16 comprises shorter chains which are no longer than a single crystalline lamella within the starch granule structure, while the longer chains of D.P. 30–100 span multiple lamellae. These two peaks replicate those observed by conventional SEC approaches for pea seed starches (Wang, Hasjim, Wu, Henry, & Gilbert, 2014). The barley starch amylopectin CLD shows a more complex structure with a major peak at D.P. 13, a shoulder at D.P. 21 and a further peak at D.P. 44. The peaks at D.P. 13 and D.P. 44 are similar to those observed in the pea CLD, but the shoulder at 21 D.P. is a distinct feature of the barley CLD, which can also be observed in the number distribution in Fig. 4a. Previous studies using HPLC-SEC derived total barley CLD’s (Gous et al., 2015; Gous, Warren, Gilbert, & Fox, 2017; Wang et al., 2014) have shown two peaks in the amylopectin region, but with the improvements in resolution available with UPLC-SEC we are also able to show the shoulder at D.P. 22. Fig. S2 also shows this difference in resolution, for the same sample analysed by HPLC-SEC. This shoulder is a feature of the barley (and more generally cereal starch) CLDs, and has been observed in a number of previous studies using FACE (Wu et al., 2013, Wu et al., 2014), or analysis of purified amylopectins (Fredriksson, Silverio, Andersson, Eliasson, & Åman, 1998), and may contribute to structural differences between cereal starches and other (e.g. legume) starches. The amylose distribution also varies between the different samples. The barley shows a bi-modal distribution of amylose with peaks at D.P. 300 and D.P. 1300 as has previously been observed for a range of cereal starches (Wang et al., 2014). The pea distributions show much lower molecular weights for their amylose fraction, as can been observed in the number distribution shown in Fig. 4b. Cultivar Cameor has a large peak at 300 D.P., similar to the barley starch, but no second peak above 1000 D.P., while BC1/19RR has only a small peak at D.P. 300 and the majority of its amylose is below this molecular weight. While amylose is an important determinant of end use quality in starch, the biosynthetic origins of structural variation in amylose fine structure are not well understood (Wang et al., 2014). These results add support to previous authors suggestions that amylose biosynthesis is a regulated process, with structural variations between varieties (Wang et al., 2014).

With the small particle size, narrow bore columns and elevated pressures used in UPLC-SEC, there is increased likelihood of both column overloading and shear scission of polymers occurring due to the elevated pressure in the column. To identify if either of these effects were giving rise to artefacts in our results we re-analysed Tipple at three different injection concentrations (0.2, 0.5 and 1 mg/mL) and at three different flow rates (150, 200 and 300 μL/min).

**Fig. 5:**
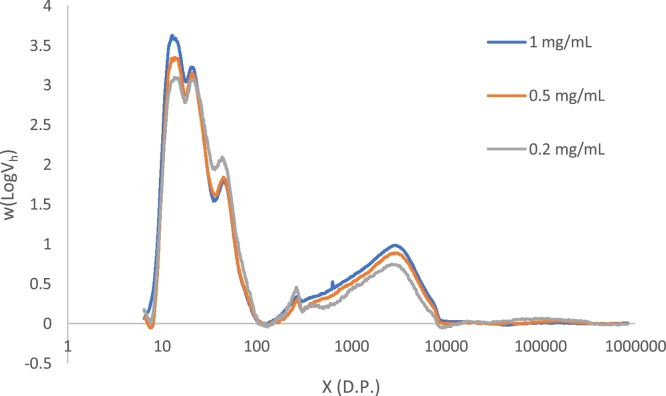
Chain Length Distribution of Tipple at three different injection concentrations, all analysed at a flow rate of 200 μL/min.

Fig. 5 shows the CLD for barley starch (Tipple) at three different injection concentrations. The distributions have been normalised using Standard Normal Variate (SNV) normalisation (Barnes, Dhanoa, & Lister, 1989; Fearn, Riccioli, Garrido-Varo, & Guerrero-Ginel, 2009) to correct for differences in signal intensity due to the differences in injection concentration. The three CLD’s closely overlap each other, with all of the same features present in each CLD, eluting at matching positions. At 0.2 mg/mL there is increased noise due to the sensitivity limits of the DRI detector, but all of the main features of the CLD are still clearly visible.

**Fig. 6:**
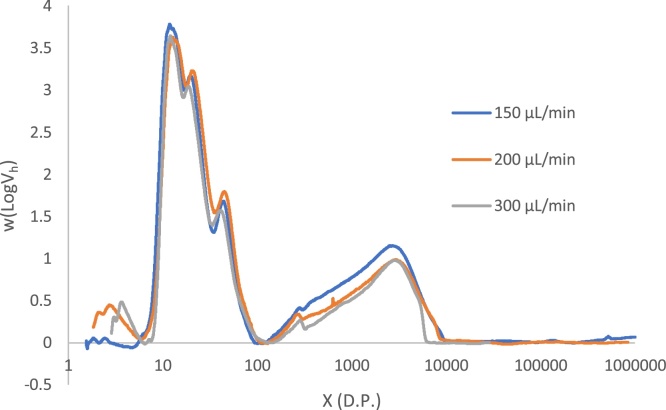
Chain Length Distribution of Tipple measured at three different flow rates, at an injection concentration of 1 mg/mL.

The effect of flow rate on possible shear scission was also investigated by running injections at a range of flow rates. The results are shown in Fig. 6. These data have also been pre-processed with SNV normalisation to correct for differences in detector response at different flow rates. From these distributions there is very limited evidence for any effect of shear reducing molecular weight at high flow rates. Linear starch polymers, such as the debranched samples used in this study, are quite resistant to the effects of shear, unlike branched amylopectin (Cave et al., 2009). This is a slight evidence of a loss of the longest amylose chains at around D.P. 10000 at 300 μL/min flow rate, but this is a relatively insignificant effect.

## 4 Conclusions

The results presented in this paper demonstrate that UPLC-SEC can be successfully applied for the rapid and efficient separation of biopolymers. Despite the very high pressures, and consequently shear rates, involved, CLDs produced by the system match very well with the molecular weights and distributions observed in a conventional HPLC-SEC set-up. The resolution obtained in terms of structural features within both the amylose and amylopectin regions is comparable or superior to existing approaches such as HPAEC-PAD, AF^4^ or FACE with no limitations on the molecular weight range that can be analysed, no requirement for derivatization, and advantages of speed. The UPLC-SEC method presented here opens up the possibilities to carry out high-throughput phenotypic screening of starch structures, using germplasm collections to identify biodiversity, to correlate phenotypes with genomic and genetic data linked to variation in the underlying genes, and to facilitate breeding programmes aimed at specific food and feed uses. The method can also be applied to other biopolymers in applications where rapid determination of molecular weight distribution is beneficial. In conclusion, we present a rapid, flexible method for biopolymer molecular weight characterisation, particularly applicable to starch CLD, which overcomes many of the technical limitations of previous methods.
