# Supplementary material for: Ultra-high performance liquid chromatography-size exclusion chromatography (UPLC-SEC) as an efficient tool for the rapid and highly informative characterisation of biopolymers
Source: Carbohydr Polym. 2018 Sep 15;196:422–6. doi: 10.1016/j.carbpol.2018.05.049 (PMC6030444; doi:10.1016/j.carbpol.2018.05.049)
Supplement: Supplementary file 1 [file mmc1.docx]

**Supplementary information: Ultra-high performance liquid chromatography-size exclusion chromatography (UPLC-SEC) as an efficient tool for the rapid and highly informative characterisation of biopolymers**

Natalia Perez^1^, Jean-Michel Plankeele^2^, Claire Domoney^3^ & Frederick J. Warren^1*^

1. Food and Health Programme, Quadram Institute Biosciences, Norwich, NR4 7UA, UK

2. Waters S.A.S., BP 608, 78056 Saint-Quentin, En Yvelines Cedex, France

3. Department of Metabolic Biology, John Innes Centre, Norwich Research Park, Colney, Norwich NR4 7UH, UK

* Corresponding author: Frederick J Warren: [fred.warren@quadram.ac.uk](mailto:fred.warren@quadram.ac.uk)

**Experimental**

Samples of debranched starches and standards, as described in the paper, were also analysed through use of a HPLC-SEC system for comparison. As described in the paper, all samples and standards were dissolved in DMSO containing 0.5% (w/v) LiBr prior to analysis at a concentration of 1 mg/mL. Samples were analysed on a Dionex ICS-3000 HPLC system fitted with a Shodex RI-71 refractive index detector and equipped with a GRAM pre-column, GRAM 30Å column and GRAM 3000Å column in series. The column temperature was held at 70°C and the samples were analysed at a flow rate of 0.5 mL/min.

**A**

**B**

Figure S1. Example elution profiles for pullulan standards (A) and example standard curve (B) derived from the retention times of the pullulan standards analysed using HPLC-SEC

Figure S2: Chain Length Distributions for three different starches expressed as weight distributions as a function of degree of polymerization, derived from HPLC-SEC elution profiles
